# Loco-Regional Treatments for Hepatocellular Carcinoma in People Living with HIV

**DOI:** 10.3390/idr14010006

**Published:** 2022-01-07

**Authors:** Cristina Micali, Ylenia Russotto, Grazia Caci, Manuela Ceccarelli, Andrea Marino, Benedetto Maurizio Celesia, Giovanni Francesco Pellicanò, Giuseppe Nunnari, Emmanuele Venanzi Rullo

**Affiliations:** 1 Unit of Infectious Diseases, Department of Clinical and Experimental Medicine, University of Messina, 98124 Messina, Italy; ylenia.russ@gmail.com (Y.R.); grazia.caci15@gmail.com (G.C.); giuseppe.nunnari@unime.it (G.N.); evenanzirullo@gmail.com (E.V.R.); 2 Unit of Infectious Diseases, Department of Clinical and Experimental Medicine, University of Catania, 95131 Catania, Italy; manuela.ceccarelli86@gmail.com (M.C.); andreamarino9103@gmail.com (A.M.); bmcelesia@gmail.com (B.M.C.); 3 Unit of Infectious Diseases, Department of Biomedical, Dental, Morphological and Functional Imaging Sciences, University of Messina, 98124 Messina, Italy; 4 Unit of Infectious Diseases, Department of Adult and Childhood Human Pathology “Gaetano Barresi”, University of Messina, 98124 Messina, Italy; giovanni.pellicano@unime.it

**Keywords:** HIV, hepatocellular carcinoma, HCC, loco-regional treatments, curative treatments, palliative treatments

## Abstract

Hepatocellular carcinoma (HCC) accounts for approximately 75–90% of primary liver cancers and is the sixth most common cancer and the third leading cause of cancer-related deaths worldwide. In the HIV-positive population, the risk of HCC is approximately four times higher than in the general population, with higher cancer-specific mortality than in HIV-negative patients. In most cases, HCC diagnosis is made in patients younger than the HIV-negative population and in the intermediate-advanced stage, thus limiting the therapeutic possibilities. Treatment choice in HIV-positive patients with HCC is subject to cancer staging, liver function and health status, as for HIV-negative and non-HIV-negative HCC patients. There are relatively few studies on the efficacy and safety in HIV-positive patients to date in loco-regional treatments for HCC. So far, literature shows that curative treatments such as radiofrequency ablation (RFA) have no significant differences in overall survival between HIV-positive and HIV-negative patients, as opposed to palliative treatments such as TACE, where there is a significant difference in overall survival. Although it can be assumed that the most recently discovered loco-regional therapies are applicable to HIV-positive patients with HCC in the same way as HIV-negative patients, further studies are needed to confirm this hypothesis. The purpose of our review is to evaluate these treatments, their efficacy, effectiveness, safety and their applicability to HIV-positive patients.

## 1. Introduction

Hepatocellular carcinoma (HCC) is one of the most common liver diseases, and it occurs as frequently as fibrosis, alcoholic liver disease, non-alcoholic fatty disease and viral hepatitis [1]. It is a malignant liver neoplasm characterized by a poor prognosis and a high metastasis rate [1,2,3].

Surgical resection is the first-line curative option for resectable HCC, while locoregional therapies (LRTs) are the main means for the clinical treatment of unresectable HCC [4,5].

People living with HIV (PLWH) show a higher risk of developing numerous chronic pathologies, such as autoimmune and tumoral diseases [6,7,8,9].

Despite PLWH being periodically monitored with HIV viral load and CD4+ lymphocyte cell count [10], they are not as frequently screened for cancers [11,12].

Frequently, HIV infection diagnosis is concomitant to another health problem, such as opportunistic infections, which poorly affect the prognosis themselves, and more often, cancers are diagnosed at an advanced stage [13,14,15].

For this reason, primary prevention is an important weapon in PLWH, in order to prevent the development of neoplasia. Primary prevention includes interventions related to lifestyle, such as avoiding alcohol consumption; sexual habits, such as more consistent condom use; and vaccination against viruses responsible for neoplastic development as Hepatitis B Virus (HBV) and Human Papillomavirus (HPV) [16,17].

With this review we aim to evaluate the efficacy, effectiveness and safety of loco-regional treatments for HCC in PLWH.

## 2. Epidemiology

HCC represents about 75–90% of primary liver tumors and is the sixth most prevalent cancer and the third leading cause of cancer-related deaths worldwide [18,19]. HCC has a strong male prevalence, with a male-to-female ratio that varies between 2:1 and 4:1 and has an unbalanced geographical distribution, with the highest incidence rates in East Asia and sub-Saharan Africa, due to the variable prevalence of the underlying risk factors. Europe ranks second after Central-Southern Asia for the lowest incidence rates of HCC, with a percentage of around 10% [20,21].

About 90% of HCC cases develop in patients with chronic liver disease, and in most cases, on a liver that has become cirrhotic. Cirrhosis is in fact the main risk factor for the development of HCC and can be determined by different causes. Overall, 54% of HCC cases are due to HBV infection, 31% to hepatitis C virus (HCV) infection and 15% are related to other causes such as alcoholism, aflatoxin exposure and non-alcoholic fatty liver disease (NAFLD) [22].

The implementation of childhood HBV vaccination programs in East Asian countries and the implementation of such programs in Asia and sub-Saharan Africa could result in a significant decrease in the HBV-infected population and therefore a significant reduction in the incidence of HBV-related HCC [23,24,25]. Despite the impossibility of primary prevention strategies for HCV-related HCC, antiviral therapies seem effective in HCV-positive patients with active infection—if not in erasing the risk, at least in reducing the incidence of HCC [26,27]. Alcohol consumption, NAFLD and non-alcoholic steatohepatitis (NASH) are risk factors for the development of HCC with an increasing relevance [28]. Other less common causes of cirrhosis contribute to a risk of developing HCC, including primary biliary cirrhosis, autoimmune hepatitis and hemochromatosis [29,30,31,32]. In some regions of Asia and Africa, exposure to aflatoxin B1, due to fungal contamination of staple foods, is related to the development of mutations (TP 53 codon 249, known as TP53 R249S) that lead to the development of HCC in patients with HBV infection [21,22,28,33].

## 3. Screening and Diagnosis

Both serological and radiological tests can be used for HCC surveillance. Serological tests include alpha-fetoprotein (AFP), des-gamma-carboxy prothrombin (DCP) or prothrombin induced by the absence of vitamin K II (PIVKA II), glycosylated AFP (L3 fraction) and total AFP ratio, alpha-fucosidase and glypican. Of these, AFP is the most used marker, although it is most commonly used as a diagnostic marker rather than for surveillance. As a matter of fact, persistently high AFP levels are a risk marker for the development of HCC. Therefore, it has been considered to exploit AFP to define populations at risk. However, as a surveillance test AFP has a suboptimal performance for two reasons: firstly, because the fluctuating levels of this marker in cirrhotic patients can be related to different situations, such as flare-ups of HBV or HCV-related infection, exacerbation of an underlying liver disease or development of HCC; secondly, because only 10–20% of tumors in the early growth phase cause abnormal levels of AFP in the serum [22].

Similarly, the use of the other serum markers has been taken into consideration more for prognosis than surveillance. In fact, it has been noted that the levels of DCP are positively related with the invasion of the portal vein and therefore with an advanced tumor stage, making this marker unsuitable for early diagnosis of HCC. The same situation has been noted regarding the AFP-L3 fraction levels [22].

In middle- and high-income countries, ultrasound (US) is the most widely used imaging method for the surveillance of patients at risk of HCC. Since HCC develops in a cirrhotic liver in 90% of cases, it also serves to monitor other conditions, such as the development of portal hypertension and the presence of ascites or thrombosis of the portal vein. The growth rate of the tumor to the limit of its own US detectability and the incidence of HCC in the population at risk are the determinants of the surveillance interval. Based on current knowledge, the guidelines show an interval of six months [22].

In HIV-positive patients, an interval of US surveillance at 6 months, however, leads to worse results regarding early diagnosis of HCC. Therefore, it reduces the chances of survival, especially in patients with an already cirrhotic liver [34]. In fact, it is known that HIV-positive patients arrive at the diagnosis of HCC at a younger age in an intermediate-advanced stage compared to the general population and that they have a clinically more aggressive course—reasons that restrict the choice of practicable therapeutic options [35,36]. Closer surveillance of HCC would therefore be largely warranted in HIV-positive patients to increase the chances of a better outcome [11], particularly if co-infected with HBV [37]. Furthermore, a low percentage of HIV patients adhere to cancer screening programs, often not sufficiently promoted by the doctor as a mandatory part of the patient’s clinical management [12,38].

## 4. HCC in HIV Patients

Sahasrabuddhe et al. [39] analyzed population data from the US HIV/AIDS Cancer Match Study (1980–2009) to assess the risk of HCC among individuals with AIDS in the USA. Out of 615,150 HIV patients, they found a risk of HCC that was nearly four times higher than the risk in the general population. Furthermore, in HIV patients, the excess risk of HCC has remained almost constant despite the progressive improvement of antiretroviral therapies from monotherapy/dual therapy in the early 1990s to the highly active therapy of today. The progress in therapies has increased the survival rates of PLWH who can now reach older ages but also have as a result a higher incidence of malignancies, especially of non-AIDS-defining cancers (NADCs) [40,41,42]. On the one hand, the incidence of AIDS-defining cancers (ADCs) such as Kaposi Sarcoma [43,44] and non-Hodgkin lymphoma [45] has decreased over time [46,47,48,49], as well as of other tumors such as Hodgkin lymphoma [50], colorectal, breast and prostate cancer [51,52,53,54,55], while on the other hand, the incidence of melanoma, anal, lung, bladder, cervix, head and neck and liver cancers has increased [56,57,58,59,60,61,62,63,64].

Cancers are a rather frequent comorbidity in PLWH and represent the main cause of death in the HIV-positive population in France [65]. In general, HIV-positive patients are diagnosed with cancer at a higher rate than the general population. They appear to have higher cancer-specific mortality than HIV-negative patients, due to diagnostic delay. In PLWH HCC nearly always reveals itself at an advanced stage of disease with limited therapeutical possibilities [66,67]. In most cases, HCC has a faster clinical presentation in PLWH and at a younger age than the general population, resulting in a shorter survival [34,37,68]. Furthermore, the overall mortality in patients with both HIV and cancer was significantly higher than expected on the basis of mortality rates for each disease separately [69].

HIV-positive patients are often simultaneously infected with HBV and/or HCV since the transmission paths are the same, and these are extremely widespread viruses that infect millions of people around the world, with higher prevalence of co-infections between people injecting drugs (PWID) and sex workers (SW) [70,71].

HIV-positive patients co-infected with HCV or HBV/HCV have been found to have a higher rate of hepatotoxicity than mono-infected HIV patients and a higher rate of progression to End Stage Liver Disease (ESLD) and HCC [35,72,73,74,75].

In cases of HCC in patients with HCV it is interesting to note that co-infection with HIV does not appear to have an impact on patient survival after diagnosis. A multicenter cohort study in 2018 found that although overall survival in HIV/HCV patients is lower compared to mono-infected patients, this condition appears to be related to lower early detection rates of HCC in HIV/HCV patients [76]. Not unlike the general population in the PLWH non-black/non-Hispanic race, diabetes, excessive alcohol consumption and a higher FIB-4 (a non-invasive index of liver fibrosis) are risk factors for developing HCC. Added to these, a low CD4 T cell count and poor viral suppression are determinants of liver complications, independent of traditional HCC risk factors. The onset of hepatic complications and hepato-related mortality is closely associated with CD4 count. It is still unclear whether this correlation is due to immunosuppression or whether a low CD4 count is itself a marker of advanced liver disease, as portal hypertension induced by cirrhosis can result in a reduced number of CD4 via splenic sequestration. However, the initiation of ART and the maintenance of suppression of the HIV viral load reduce the risk of ESDL and HCC in HIV-positive patients [72,77,78,79,80].

Furthermore, among PLWH with untreated HCC, an undetectable HIV viral load seems to be an independent predictor of better survival [81].

## 5. Staging and Treatments

For HCC treatment, several options are available. The choice relies upon the stage of the cancer and liver function at the time of diagnosis. According to the current guidelines of the European Association for the Study of the Liver (EASL), treatment choice is based on the staging of HCC, defined by a modified version of the Barcelona Cancer Liver Center (BCLC) algorithm. In modified BCLC, the Child–Pugh classification for evaluation of liver function was eliminated [22].

BCLC identifies five different stages of HCC: very early (0), early (A), intermediate (B), advanced (C) and end stage (D). Prognostic prediction is based on variables related to tumor status, such as size; number of nodules; vascular invasion; liver function, such as bilirubin levels; presence of portal hypertension; liver function preservation; and health status as defined by the Eastern Cooperative Oncology Group (ECOG) [22].

ECOG is a performance status scale based on five worsening degrees: patient fully active and completely able to carry on all pre-disease performance without restriction (grade 0); patient with restricted physically strenuous activity but ambulatory and able to carry out work of a light or sedentary nature (grade 1); patient ambulatory and capable of all self-care but unable to carry out any work activities (grade 2); patient capable of only limited self-care, confined to bed or chair for more than 50% of waking hours (grade 3); patient completely disabled and unable to carry out any self-care, confined to bed or chair at all times (grade 4); patient dead (grade 5) [82].

Very early HCC (BCLC stage 0) is defined as the presence of a single nodule smaller than < 2 cm in diameter without vascular invasion/satellites in patients with good health status (ECOG-0) and well-preserved liver function (Child–Pugh A class). Patients with very early-stage HCC who undergo resection have a 5-year survival rate of 80–90%. The complete tumor necrosis that can be achieved with radiofrequency ablation (RFA) has made this technique equally usable in this stage, with similar results in terms of survival as well as expectation and quality of life [22].

Early HCC (BCLC stage A) is characterized by the presence of a single nodule > 2 cm or three nodules < 3 cm in diameter, ECOG-0 and preserved liver function. Five years after curative treatment (resection, RFA, hepatic transplant), the average survival rate of patients in this stage is 50–70%; the choice of treatment is based on the so-called treatment-dependent variables. For example, the state of the tumor in terms of size and multicentricity affects the choice of treatment and consequently its result. For a single lesion > 5 cm in diameter, surgical resection is still considered the option of choice. In this case, variables related to liver function, such as the absence of clinically relevant portal hypertension and normal bilirubin levels are key predictors of survival. Similarly, tumor status is a key variable in liver transplant candidates, who must have a single nodule ≤ 5 cm or three nodules ≤ 3 cm. Otherwise, in patients undergoing local ablation, Child–Pugh class A is the most important prognostic variable, along with tumor size [22].

Intermediate HCC (BCLC stage B) is characterized by the presence of asymptomatic multinodular tumors without vascular invasion or extrahepatic diffusion. The median survival rate in untreated patients is 49% at two years. The first-choice therapeutic approach is represented by trans-arterial chemoembolization (TACE) [22].

Advanced HCC (BCLC stage C) refers to patients with cancer-related symptoms, ECOG 1-2, macrovascular invasion or extrahepatic spread (involvement of the lymph nodes or metastasis). Prognosis is poor, with an expected median survival time of 6–8 months and a one-year survival rate of 25%. Over the past decade, the introduction of sorafenib, a tyrosine kinase inhibitor, has led to a dramatic improvement in survival in patients with advanced HCC, also paving the way for the research of other targeted agents (regorafenib, lenvatinib, cabozantinib) [22].

End-stage HCC (BCLC stage D) includes patients with end-stage disease, ECOG 3-4, caused by severe cancer-related disability. Median survival is 3–4 months and 11% at one year [4]. For these patients, there are currently no therapeutic options available, so only supportive therapy is recommended [83].

Concerning medical treatments with kinase inhibitors as sorafenib, HIV infection was for many years considered an exclusion criterion in large phase III trials [84] as well as in liver transplantation [85].

In the past few years, four cases have reported data about co-administration of sorafenib and highly active antiretroviral therapy in PLWH with HCC [86,87,88,89]. In one case, therapeutic drug monitoring performed during co-administration of sorafenib showed a concentration of fosamprenavir under the minimum level recommended by international guidelines, suggesting a possible interaction [88]. Instead, in two cases it was observed that the simultaneous administration of sorafenib and antiretroviral therapy was well tolerated [86,87]. However, these cases remain still anecdotal.

The first study about safety and efficacy of sorafenib in PLWH with unresectable HCC in concomitant highly active antiretroviral therapy is an Italian retrospective study. It shows favorable survival data among PLWH with HCC treated with sorafenib, together with a reasonable safety profile. [90].

To date, no data are available about the use of other target agents as regorafenib, lenvatinib or cabozantinib in PLWH with HCC. Moreover, further trials specifically designed for PLWH and different strategies to integrate HIV and HCC treatments are necessary.

## 6. Loco-Regional Treatments

In the field of interventional oncology, numerous minimally invasive treatments have been developed, including curative and palliative options. The curative treatments are percutaneous radiofrequency ablation (RFA), microwave ablation (MWA), percutaneous ethanol injection (PEI), cryoablation (CA) and irreversible electroporation (IRE). The palliative treatments are trans-arterial chemoembolization (TACE), transcatheter arterial chemotherapy infusion (TACI) and, more recently, selective internal radiation therapy (SIRT) [18].

### 6.1. Curative Loco-Regional Treatments

When HCC is diagnosed in the earliest stages, it is possible to intervene with less invasive procedures than surgical ones. Over time, various techniques based on localized tumor destruction have been developed by exploiting physically or chemically induced cell necrosis [22].

The technique that opened this path was the PEI, a method capable of causing coagulative necrosis of the neoplasm through cell dehydration, denaturation of proteins and chemical occlusion of the small vessels that supply the tumor. Although percutaneous injection of ethanol results in complete necrosis in 90% of tumors < 2 cm, it leads to incomplete necrosis in most larger tumors, with local recurrence rates of up to 49%. Furthermore, once injected into the lesion, the distribution of ethanol does not pass beyond the cirrhotic fibrous tissue surrounding the HCC. In this regard, a very similar technique, based on the percutaneous injection of acetic acid, has not shown substantial advantages over PEI [22].

In contrast, numerous studies have shown the superiority of RFA compared to PEI in terms of overall survival, disease-free survival and recurrence rate. RFA is part of thermo-ablative therapies, procedures subsequently developed, which induce tumor necrosis by causing tissue heating by radio frequency (RFA) or microwaves (MWA) or, on the contrary, causing freezing (CA) [22].

The different physical phenomenon underlying the heat generated is the determinant of the differences between RFA and MWA. Despite the technical similarities, MWA presents a better convection profile, generates constant higher intra-tumoral temperatures, offers faster ablation times and, most importantly, the ability to treat multiple lesions simultaneously with multiple probes [91]. In patients with a single small nodule (≤3 cm) located near the great vessels (portal vein and hepatic vein), MWA appears to have better control over tumor progression than RFA [92,93].

CA is a safe and effective ablative technique on par with RFA in early or very early-stage HCC and in small HCC with perivascular localization [94]. Additionally, complications such as vascular thrombosis and hepatic infarction occur less frequently than in RFA [95]. In subcapsular HCC, the use of RFA has been questioned—not only because this localization affects the success of ablation, posing the risk of local tumor progression, but also because of the greater possibility of complications during and after the procedure. Although some of these problems could be solved by resorting to the use of artificial ascites or by performing laparoscopic ablation, in some cases it is not feasible to safely conduct RFA in subcapsular tumors, especially in certain circumstances such as proximity to the gallbladder. Since CA generates ice balls whose formation can be monitored in real time via ultrasound, CT or MRI, and because the ablation area can be controlled, this procedure can be selected to treat tumors in the vicinity of the gallbladder, main bile ducts, diaphragm and other high-risk sites as sub-cardiac localization [96,97,98].

IRE is part of the ablation techniques, but it uses high-current electrical pulses to induce pore formation in the cancer cell membrane, causing necrosis [22]. It is an effective technique in early-stage HCCs that are neither resectable nor eligible for standard thermal-ablative procedures, thanks to the ability to preserve blood vessels and bile ducts which are particularly relevant for tumors that are centrally placed [99,100,101,102]. Especially for smaller lesions, it appears to have excellent complete response rates and low local recurrence rates [103]. To date there are few comparative studies in the literature between this technique and the thermo-ablative ones. However, it seems that IRE has a post-procedural complication rate comparable to that of MWA/RFA, despite the higher number of punctures and the lack of track cauterization in IRE [104]. Conversely, IRE has an earlier reparative process in the ablation area with consequent earlier shrinkage of the area itself, compared to RFA, appreciable in contrast-enhanced ultrasound (CEUS) [105,106]. Moreover, compared to RFA/MWA, IRE is a more difficult method to deliver the treatment, and is more expensive since it requires general anesthesia to ensure blockage of the musculature during the procedure, as stimulation induced by electrical impulses causes muscle contraction [22].

### 6.2. Palliative Loco-Regional Treatments

In patients who cannot undergo liver transplantation, surgical resection or percutaneous ablative procedures, available treatment options include loco-regional palliative treatments such as SIRT, TACE and TACI.

SIRT or trans arterial radio-embolization (TARE) is a loco-regional treatment used in intermediate or advanced stages of HCC (BLCL–B or C), in patients who cannot undergo TACE nor tolerate treatment with sorafenib [18]. SIRT consists in the introduction of radioactive substances through an intra-arterial catheter, positioned by means of radiographic guidance, in order to deliver a tumoricidal dose of radiation to the target neoplasm, saving the adjacent healthy liver parenchyma. The location of the catheter in the common hepatic artery, in the right or left hepatic artery or in the smaller branches depends on the size of the tumor. The most widely used radionuclide is represented by yttrium-90 marked on resin microspheres (SIR-Spheres) or embedded in glass microspheres (TheraSpheres™). The size of the microspheres (20–60 µm) allows a peritumoral vascular arrangement, preventing the passage into the venous circulation: they remain in fact confined in the liver and are neither metabolized nor excreted [106].

Transcatheter arterial chemoembolization (TACE) is considered the gold standard in patients classified as BCLC-B: patients not eligible for surgery or percutaneous ablation procedures, who have preserved liver function, no vascular invasion nor extrahepatic spread. It is based on the occlusion of the arterial flow that supplies the target neoplastic lesion, by embolizing microparticles, combined with the injection of chemotherapeutic drugs in such a way as to spare the adjacent healthy liver tissue [18].

Some studies in the past [107,108] have shown that TACE in patients with intermediate or advanced HCC is associated with longer survival than TACI. In the current literature [109] it is found that the TACE is a safer procedure and able to determine a longer survival in patients with HCC not eligible for surgical treatment.

Several comparative studies have been conducted between TACE and SIRT in patients with HCC at various BCLC stages. In the retrospective study of Soydal and others [110], for the same safety and efficacy the two treatments differ in the overall survival length, which is 30.63 ± 3.68 months for the group of patients undergoing SIRT and 39.24 ± 4.62 months for the group treated with TACE. In contrast, in the study of Gordon et al. [111] there was no statistically significant difference in overall survival between patients treated with TACE and patients treated with SIRT, but there was a better local control of tumor and a lower drop-out from the transplant list in patients undergoing SIRT. Another study [112] also found a similar impact in the two treatments on health-related quality of life (HRQoL).

### 6.3. Loco-Regional Treatments in HIV Patients

Unlike cancers as non-small cell lung, anal cancer, Hodgkin’s lymphoma and cervical cancer, for which in 2018 an attempt was made to fill the lack of appropriate guidelines on the management and treatment of PLWH affected by these neoplasms [113], to date there are no guidelines for the management of HIV-positive patients with HCC. Several studies have allowed us to evaluate over time the efficacy and safety of available treatments, such as liver transplantation [85,114,115] or surgical resection [116,117], or the most recent loco-regional techniques. In our best knowledge, although methods such as SIRT, MWA, CA and IRE have been evaluated in the general population, the current literature on HIV-infected HCC patients is missing. There are still no studies to verify their efficacy in the HIV-positive population and to compare their safety, outcome and post-procedural complications with the general population.

Conversely, several studies investigated the performance of RFA and TACE in HIV-positive HCC patients, considering only liver function and tumor stage at the time of treatment.

Kong et al. [118] conducted a control-case study on a total of 2249 patients with BCLC-B stage HCC, dividing them into three groups and analyzing prognostic and survival factors. The three groups consisted of 21 HIV-positive patients who received TACE treatment (HIV+ TACE+), 1293 HIV-negative patients treated with TACE (HIV-TACE+) and 150 HIV-negative patients who did not undergo TACE (HIV-TACE-). After 1:2 matching, the one- and two-year survival rate of HIV+ TACE+ and HIV- TACE+ groups was 64.3% and 76.5% (*p* = 0.453) and 45.5% vs. 50.0% (*p* = 0.790), respectively. Furthermore, by comparing one- and two-year survival rates between HIV+ TACE+ and HIV-TACE- it was found that the one-year overall survival rate was 64.3% vs. 45.7% (*p* = 0.097) and the two-year survival rate was 45.5% vs. 7.1% (*p* = 0.004).

The study by Lim et al. [119] came to the same conclusion that HIV status does not have a statistically significant impact on prognosis, and that HIV patients could therefore share the same therapeutic opportunities as HIV-negative patients. They evaluated 473 new HCC patients, 450 of whom were HIV-negative (HIV-) and 23 (4.9%) of whom were HIV-positive (HIV+). The control group of HIV- was matched with HIV+ for some prognostic variables such as etiology, severity of the underlying liver disease, the BCLC stage, tumor extension and the year of diagnosis. Ten HIV+ patients received curative treatment (liver transplantation, surgical resection) and six of them underwent RFA, while thirteen HIV+ received palliative treatment (supportive care, chemotherapy) and seven of them underwent TACE. The HIV+ and HIV- patients did not differ significantly in terms of the 1-year survival rate (65% vs. 76%, respectively) or the 3-year survival rate (44% vs. 48%, respectively).

Similarly, the study conducted by Berretta et al. [120], by comparing 95 HCC HIV+ with 338 HCC HIV- and stratifying according to the treatment administered, showed a survival time of 51 months and 53 months, respectively for HIV-infected and uninfected patients undergoing RFA/PEI curative treatment. In contrast, the study highlighted a statistically significant difference in survival between HIV+ and HIV- patients of 35 and 65 months, respectively, when undergoing palliative treatment such as TACE.

## 7. Conclusions

HCC, as with other non-AIDS-defining cancers, increasingly affects the quality of life and life expectancy of HIV+ patients. In most cases, the delay in diagnosis and the presentation of the tumor in the medium-to-advanced stage are the main factors that determine a poor prognosis and a lower chance of survival compared to the general population. The implementation of screening programs, aimed at a shorter time interval in the execution of control ultrasound scans and to promote greater adherence to these programs, also through psychological support, could allow the diagnosis of HCC at an early or even very early stage. Treatment options for HCC in HIV+ patients are subject to tumor staging, liver function and health status, as for HIV- patients affected by HCC, since HIV status has no influence on treatment choice. Although it can be assumed that the most recently discovered loco-regional therapies are applicable to HIV-positive patients with HCC, further studies are needed to confirm this hypothesis.

To summarize, the studies conducted so far seem to demonstrate that HIV status does not prevent HIV patients from being subjected to the same treatments as the general population with the same stage stratification according to the BCLC algorithm. If a curative treatment such as RFA does not show significant differences in overall survival between the two populations, it appears that such a difference may exist when it comes to palliative treatments. In any case, further and more extensive studies are certainly needed to investigate the real possibilities of loco-regional treatments for HIV-positive patients.

## Data Availability

No appliable.

## References

[1] Ali A.L., Nailwal N.P., Doshi G.M. (2021). Emerging Role of Interleukins for the Assessment and Treatment of Liver Diseases. Endocrine Metab. Immune Disord. Drug Targets.

[2] Yeganeh H.H., Heiat M., Kieliszek M., Alavian S.M., Rezaie E. (2021). DT389-YP7, a Recombinant Immunotoxin against Glypican-3 That Inhibits Hepatocellular Cancer Cells: An In Vitro Study. Toxins.

[3] Fu X.-W., Song C.-Q. (2021). Identification and Validation of Pyroptosis-Related Gene Signature to Predict Prognosis and Reveal Immune Infiltration in Hepatocellular Carcinoma. Front. Cell Dev. Biol..

[4] Delvecchio A., Conticchio M., Riccelli U., Ferraro V., Ratti F., Gelli M., Anelli F.M., Laurent A., Vitali G.C., Magistri P. (2021). Laparoscopic versus open liver resection for hepatocellular carcinoma in elderly patients: A propensity score matching analysis. HPB.

[5] Xue J., Ni H., Wang F., Xu K., Niu M. (2021). Advances in locoregional therapy for hepatocellular carcinoma combined with immunotherapy and targeted therapy. J. Interv. Med..

[6] Pinzone M.R., Ceccarelli M., Rullo E.V., Maresca M., Bruno R., Condorelli F., Di Rosa M., Madeddu G., Foca’ E., Calcagno A. (2019). Circulating angiopoietin-like protein 2 levels are associated with decreased renal function in HIV+ subjects on cART: A potential marker of kidney disease. Biomed. Rep..

[7] Pellicano C., Leodori G., Innocenti G.P., Gigante A., Rosato E. (2019). Microbiome, Autoimmune Diseases and HIV Infection: Friends or Foes?. Nutrients.

[8] Noubissi E.C., Katte J.-C., Sobngwi E. (2018). Diabetes and HIV. Curr. Diabetes Rep..

[9] Roszkiewicz J., Smolewska E. (2016). Kaleidoscope of autoimmune diseases in HIV infection. Rheumatol. Int..

[10] Celesia B.M., Marino A., Del Vecchio R.F., Bruno R., Palermo F., Gussio M., Nunnari G., Cacopardo B. (2019). Is it safe and cost saving to defer the cd4+ cell count monitoring in stable patients on art with more than 350 or 500 cells/µL?. Mediterr. J. Hematol. Infect. Dis..

[11] D’Andrea F., Ceccarelli M., Rullo E.V., Facciolà A., d’Aleo F., Cacopardo B., Iacobello C., Costa A., Altavilla G., Pellicanò G.F. (2018). Cancer screening in HIV-infected patients: Early diagnosis in a high-risk population. WCRJ.

[12] Ceccarelli M., Condorelli F., Venanzi Rullo E., Pellicanò G.F. (2018). Editorial—Improving access and adherence to screening tests for cancers: A new, though old, challenge in the HIV epidemics. World Cancer Res. J..

[13] Celesia B.M., Marino A., Borracino S., Arcadipane A.F., Pantò G., Gussio M., Coniglio S., Pennisi A., Cacopardo B., Panarello G. (2020). Successful Extracorporeal Membrane Oxygenation Treatment in an Acquired Immune Deficiency Syndrome (AIDS) Patient with Acute Respiratory Distress Syndrome (ARDS) Complicating Pneumocystis jirovecii Pneumonia: A Challenging Case. Am. J. Case Rep..

[14] Marino A., Caltabiano E., Zagami A., Onorante A., Zappalà C., Locatelli M.E., Pampaloni A., Scuderi D., Bruno R., Cacopardo B. (2018). Rapid emergence of cryptococcal fungemia, Mycobacterium chelonae vertebral osteomyelitis and gastro intestinal stromal tumor in a young HIV late presenter: A case report. BMC Infect. Dis..

[15] Coghill A.E., Han X., Suneja G., Lin C.C., Jemal A., Shiels M.S. (2019). Advanced stage at diagnosis and elevated mortality among US patients with cancer infected with HIV in the National Cancer Data Base. Cancer.

[16] D’Andrea F., Ceccarelli M., Rullo E.V., Facciolà A., Marino A., Cacopardo B., Pellicanò G.F., Nunnari G. (2019). Vaccines against HPV in people living with HIV: A review. World Cancer Res. J..

[17] Jose-Abrego A. (2019). Some considerations about HBV vaccination among HIV patients from Latin America and the Caribbean. Ann. Hepatol..

[18] Inchingolo R., Posa A., Mariappan M., Spiliopoulos S. (2019). Locoregional treatments for hepatocellular carcinoma: Current evidence and future directions. World J. Gastroenterol..

[19] Forner A., Llovet J.M., Bruix J. (2012). Hepatocellular carcinoma. Lancet.

[20] Hefaiedh R., Ennaifer R., Romdhane H., Ben Nejma H., Arfa N., Belhadj N., Gharbi L., Khalfallah T. (2013). Gender difference in patients with hepatocellular carcinoma. La Tunis. Med..

[21] Singal A.G., Lampertico P., Nahon P. (2020). Epidemiology and surveillance for hepatocellular carcinoma: New trends. J. Hepatol..

[22] (2018). EASL Clinical Practice Guidelines: Management of hepatocellular carcinoma. J. Hepatol..

[23] Chang M.-H., You S.-L., Chen C.-J., Liu C.-J., Lee C.-M., Lin S.-M., Chu H.-C., Wu T.-C., Yang S.-S., Kuo H.-S. (2009). Decreased Incidence of Hepatocellular Carcinoma in Hepatitis B Vaccinees: A 20-Year Follow-up Study. JNCI J. Natl. Cancer Inst..

[24] Kao J.-H. (2015). Hepatitis B vaccination and prevention of hepatocellular carcinoma. Best Pr. Res. Clin. Gastroenterol..

[25] Amponsah-Dacosta E. (2021). Hepatitis B virus infection and hepatocellular carcinoma in sub-Saharan Africa: Implications for elimination of viral hepatitis by 2030?. World J. Gastroenterol..

[26] Kanwal F., Kramer J., Asch S.M., Chayanupatkul M., Cao Y., El-Serag H.B. (2017). Risk of Hepatocellular Cancer in HCV Patients Treated with Direct-Acting Antiviral Agents. Gastroenterology.

[27] Shiha G., Mousa N., Soliman R., Mikhail N.N., Elbasiony M., Khattab M. (2020). Incidence of HCC in chronic hepatitis C patients with advanced hepatic fibrosis who achieved SVR following DAAs: A prospective study. J. Viral Hepat..

[28] Asfari M.M., Sarmini M.T., AlOmari M., Lopez R., Dasarathy S., McCullough A.J. (2020). The association of nonalcoholic steatohepatitis and hepatocellular carcinoma. Eur. J. Gastroenterol. Hepatol..

[29] Liang Y., Yang Z., Zhong R. (2012). Primary biliary cirrhosis and cancer risk: A systematic review and meta-analysis. Hepatology.

[30] Zhang X.-X., Wang L.-F., Jin L., Li Y.-Y., Hao S.-L., Shi Y.-C., Zeng Q.-L., Li Z.-W., Zhang Z., Lau G.K. (2015). Primary biliary cirrhosis-associated hepatocellular carcinoma in Chinese patients: Incidence and risk factors. World J. Gastroenterol..

[31] Manivannan A., Mazumder S., Al-Kourainy N. (2020). The Role of Hepatocellular Carcinoma Surveillance in Autoimmune Hepatitis. Cureus.

[32] Kowdley K.V. (2004). Iron, hemochromatosis, and hepatocellular carcinoma. Gastroenterology.

[33] Han C., Yu T., Qin W., Liao X., Huang J., Liu Z., Yu L., Liu X., Chen Z., Yang C. (2020). Genome-wide association study of the TP53 R249S mutation in hepatocellular carcinoma with aflatoxin B1 exposure and infection with hepatitis B virus. J. Gastrointest. Oncol..

[34] Merchante N., Figueruela B., Rodríguez-Fernández M., Rodríguez-Arrondo F., Revollo B., Ibarra S., Galindo M.J., Merino E., Montero M., Téllez F. (2019). Low performance of ultrasound surveillance for the diagnosis of hepatocellular carcinoma in HIV-infected patients. AIDS.

[35] Berretta M., Di Francia R., Di Benedetto F., Tirelli U. (2015). New Entities in the treatment of hepatocellular carcinoma: HIV-positive and elderly patients. WCRJ.

[36] Benmassaoud A., Nitulescu R., Pembroke T., Halme A.S., Ghali P., Deschenes M., Wong P., Klein M.B., Sebastiani G. (2018). Liver-related Events in Human Immunodeficiency Virus–infected Persons with Occult Cirrhosis. Clin. Infect. Dis..

[37] Okeke E., Davwar P.M., Mullen B., Duguru M., Agbaji O., Sagay A., Murphy R., Hawkins C. (2021). The impact of HIV on hepatocellular cancer survival in Nigeria. Trop. Med. Int. Health.

[38] Ruzzenente A., Valdegamberi A., Conci S., Guglielmi A. (2016). Treatment Approach of HCC. Text Book Title Hepatocellular Carcinoma in the 3rd Millennium.

[39] Sahasrabuddhe V.V., Shiels M.S., McGlynn K.A., Engels E.A. (2012). The risk of hepatocellular carcinoma among individuals with acquired immunodeficiency syndrome in the United States. Cancer.

[40] Nozza S., Malagoli A., Maia L., Calcagno A., Focà E., De Socio G., Piconi S., Orofino G., Cattelan A.M., Celesia B.M. (2017). Antiretroviral therapy in geriatric HIV patients: The GEPPO cohort study. J. Antimicrob. Chemother..

[41] Ceccarelli M., Venanzi Rullo E., d’Aleo F., Pellicanò G.F., D’Andrea F., Marino A., Cacopardo B., Celesia B.M., La Rocca G., Di Rosa M. (2020). Non-AIDS defining cancers: A comprehensive update on diagnosis and management. Eur. Rev. Med. Pharmacol. Sci..

[42] Rullo E.V., Ceccarelli M., Condorelli F., Visalli G., D’Aleo F., Paolucci I., Cacopardo B., Pinzone M.R., Di Rosa M., Nunnari G. (2019). Investigational drugs in HIV: Pros and cons of entry and fusion inhibitors (Review). Mol. Med. Rep..

[43] D’Andrea F., Facciolà A., Coco M.G., Micali C., Paolucci I., Maranto D., Costantino M.L.P., Larnè D., Mondello P., Pellicanò G.F. (2019). Kaposi’s sarcoma and psoriasis in a naïve HIV-positive patient: A case report. Infect. Dis. Trop. Med..

[44] Ceccarelli M., Facciolà A., Taibi R., Pellicanò G.F., Nunnari G., Venanzi Rullo E. (2019). The treatment of Kaposi’s sarcoma: Present and future options, a review of the literature. Eur. Rev. Med. Pharmacol. Sci..

[45] D’Andrea F., Venanzi Rullo E., Facciolà A., Di Rosa M., Condorelli F., Marino A., Cacopardo B., Pellicanò G.F., Nunnari G., Ceccarelli M. (2020). Epstein Barr Virus related cancer in people living with HIV: A review of the literature. World Cancer Res. J..

[46] Facciolà A., Venanzi Rullo E., Ceccarelli M., D’Aleo F., Di Rosa M., Pinzone M.R., Condorelli F., Visalli G., Picerno I., Fisichella R. (2017). Kaposi’s sarcoma in HIV-infected patients in the era of new antiretrovirals. Eur. Rev. Med. Pharmacol. Sci..

[47] Ji Y., Lu H. (2017). Malignancies in HIV-Infected and AIDS Patients. Adv. Exp. Med. Biol..

[48] Casper C., Crane H., Menon M., Money D., Holmes K.K., Bertozzi S., Bloom B.R., Jha P. (2017). HIV/AIDS Comorbidities: Impact on Cancer, Noncommunicable Diseases, and Reproductive Health. Major Infectious Diseases.

[49] Berretta M., Martellotta F., Di Francia R., Spina M., Vaccher E., Balestreri L., Borsatti E., Bearz A., De Paoli P., Tirelli U. (2015). Clinical presentation and outcome of non-AIDS defining cancers, in HIV-infected patients in the ART-era: The Italian Cooperative Group on AIDS and tumors activity. Eur. Rev. Med. Pharmacol. Sci..

[50] Facciolà A., Rullo E.V., Ceccarelli M., d’Aleo F., D’Andrea F., Visalli G., Pinzone M.R., Picerno I., Cacopardo B., Condorelli F. (2019). Hodgkin’s lymphoma in people living with HIV: Epidemiology and clinical management. WCRJ.

[51] Facciolà A., Ceccarelli M., Venanzi Rullo E., d’Aleo F., Condorelli F., Visalli G., Cacopardo B., Pinzone M.R., Di Rosa M., Nunnari G. (2018). Prostate cancer in HIV-positive patients: A review of the literature. World Cancer Res. J..

[52] d’Aleo F., Venanzi Rullo E., Ceccarelli M., Facciolà A., Condorelli F., Pinzone M.R., Cacopardo B., Di Rosa M., Nunnari G., Pellicanò G.F. (2018). HIV and colorectal cancer. New insights and review of the literature. World Cancer Res. J..

[53] Sun D., Cao M., Li H., Ren J., Shi J., Li N., Chen W. (2021). Risk of prostate cancer in men with HIV/AIDS: A systematic review and meta-analysis. Prostate Cancer Prostatic Dis..

[54] Coghill A.E., Engels E.A., Schymura M.J., Mahale P., Shiels M.S. (2018). Risk of Breast, Prostate, and Colorectal Cancer Diagnoses Among HIV-Infected Individuals in the United States. J. Natl. Cancer Inst..

[55] D’Andrea F., Ceccarelli M., Facciolà A., Nunnari G., Pellicanò G.F., Venanzi Rullo E. (2019). Breast cancer in women living with HIV. Eur. Rev. Med. Pharmacol. Sci..

[56] Facciolà A., Venanzi Rullo E., Ceccarelli M., D’Andrea F., Coco M., Micali C., Cacopardo B., Marino A., Cannavò S.P., Di Rosa M. (2020). Malignant melanoma in HIV: Epidemiology, pathogenesis and management. Dermatol. Ther..

[57] d’Aleo F., Ceccarelli M., Venanzi Rullo E., Facciolà A., d’Andrea F., Micali C., Coco M., Pinzone M.R., Focà E., Condorelli F. (2019). Anal cancer in people living with HIV: The importance of the screening and of early diagnosis. WCRJ.

[58] Sigel K., Makinson A., Thaler J. (2017). Lung cancer in persons with HIV. Curr. Opin. HIV AIDS.

[59] D’Aleo F., Cama B.A.V., Paolucci I.A., Venanzi Rullo E., Condorelli F., Facciolà A., Di Francia R., Savasta A., Pinzone M.R., Picerno I. (2018). New and old assumptions on lung cancer in people living with HIV. World Cancer Res. J..

[60] Visalli G., Facciolà A., D’Aleo F., Pinzone M.R., Condorelli F., Picerno I., Nunnari G., Pellicanò G.F., Ceccarelli M., Venanzi Rullo E. (2018). HPV and urinary bladder carcinoma: A review of the literature. World Cancer Res. J..

[61] D’Andrea F., Pellicanò G.F., Venanzi Rullo E., d’Aleo F., Facciolà A., Micali C., Coco M., Visalli G., Picerno I., Condorelli F. (2019). Cervical cancer in women living with HIV: A review of the literature. World Cancer Res. J..

[62] Ceccarelli M., Rullo E.V., Facciolà A., Madeddu G., Cacopardo B., Taibi R., D’Aleo F., Pinzone M.R., Picerno I., Di Rosa M. (2018). Head and neck squamous cell carcinoma and its correlation with human papillomavirus in people living with HIV: A systematic review. Oncotarget.

[63] D’Andrea F., Venanzi Rullo E., Marino A., Moscatt V., Celesia B.M., Cacopardo B., Condorelli F., La Rocca G., Di Rosa M., Pellicanò G.F. (2020). Hepatitis B virus infection and hepatocellular carcinoma in PLWH: Epidemiology, pathogenesis and treatment. World Cancer Res. J..

[64] Robbins H.A., Shiels M.S., Pfeiffer R.M., Engels E.A. (2014). Epidemiologic contributions to recent cancer trends among HIV-infected people in the United States. AIDS.

[65] Abbar B., Veyri M., Solas C., Poizot-Martin I., Spano J.-P. (2020). VIH et cancer: Mise au point en 2020. Bull Cancer.

[66] d’Aleo F., Ceccarelli M., Venanzi Rullo E., Facciolà A., Di Rosa M., Pinzone M.R., Condorelli F., Visalli G., Picerno I., Berretta M. (2017). Hepatitis C-related hepatocellular carcinoma: Diagnostic and therapeutic management in HIV-patients. Eur. Rev. Med. Pharmacol. Sci..

[67] Coghill A.E., Shiels M.S., Suneja G., Engels E.A. (2015). Elevated cancer-specific mortality among HIV-infected patients in the United States. J. Clin. Oncol..

[68] Gelu-Simeon M., Lewin M., Ostos M., Bayan T., Delgado M.B., Teicher E., Layese R., Roudot-Thoraval F., Fontaine H., Sobesky R. (2018). Prognostic factors of survival in HIV/HCV co-infected patients with hepatocellular carcinoma: The CARCINOVIC Cohort. Liver Int..

[69] Coghill A.E., Pfeiffer R.M., Shiels M.S., Engels E.A. (2017). Excess Mortality among HIV-Infected Individuals with Cancer in the United States. Cancer Epidemiol. Biomark. Prev..

[70] Hu J., Liu K., Luo J. (2019). HIV–HBV and HIV–HCV Coinfection and Liver Cancer Development. Hematop. Growth Factors Oncol..

[71] Rashti R., Alavian S.M., Moradi Y., Sharafi H., Bolbanabad A.M., Roshani D., Moradi G. (2020). Global prevalence of HCV and/or HBV coinfections among people who inject drugs and female sex workers who live with HIV/AIDS: A systematic review and meta-analysis. Arch. Virol..

[72] Re V.L., Newcomb C.W., Carbonari D.M., Roy J.A., Althoff K.N., Kitahata M.M., Reddy K.R., Lim J.K., Silverberg M.J., Mayor A.M. (2019). Determinants of Liver Complications Among HIV/Hepatitis B Virus–Coinfected Patients. JAIDS J. Acquir. Immune Defic. Syndr..

[73] Su S., Fairley C.K., Sasadeusz J., He J., Wei X., Zeng H., Jing J., Mao L., Chen X., Zhang L. (2018). HBV, HCV, and HBV/HCV co-infection among HIV-positive patients in Hunan province, China: Regimen selection, hepatotoxicity, and antiretroviral therapy outcome. J. Med Virol..

[74] Kumar A., Acharya S.K., Singh S.P., Arora A., Dhiman R.K., Aggarwal R., Anand A.C., Bhangui P., Chawla Y.K., Gupta S.D. (2020). 2019 Update of Indian National Association for Study of the Liver Consensus on Prevention, Diagnosis, and Management of Hepatocellular Carcinoma in India: The Puri II Recommendations. J. Clin. Exp. Hepatol..

[75] Kramer J.R., Kowalkowski M.A., Duan Z., Chiao E.Y. (2015). The Effect of HIV Viral Control on the Incidence of Hepatocellular Carcinoma in Veterans with Hepatitis C and HIV Coinfection. JAIDS J. Acquir. Immune Defic. Syndr..

[76] Merchante N., Rodríguez-Fernández M., Figueruela B., Rodríguez-Arrondo F., Revollo B., Ibarra S., Téllez F., Merino E., Montero-Alonso M., Galindo M.J. (2020). Impact of HIV on the survival of hepatocellular carcinoma in hepatitis C virus-infected patients. AIDS.

[77] Torgersen J., Kallan M.J., Carbonari D.M., Park L.S., Mehta R.L., D’Addeo K., Tate J.P., Lim J.K., Goetz M.B., Rodriguez-Barradas M.C. (2019). HIV RNA, CD4+ Percentage, and Risk of Hepatocellular Carcinoma by Cirrhosis Status. J. Natl. Cancer Inst..

[78] Clifford G.M., Rickenbach M., Polesel J., Maso L.D., Steffen I., Ledergerber B., Rauch A., Probst-Hensch N.M., Bouchardy C., Levi F. (2008). Influence of HIV-related immunodeficiency on the risk of hepatocellular carcinoma. AIDS.

[79] Park L.S., Tate J.P., Justice A.C., Re V.L., Lim J.K., Bräu N., Brown S.T., Butt A.A., Gibert C., Goetz M. (2011). FIB-4 Index Is Associated with Hepatocellular Carcinoma Risk in HIV-Infected Patients. Cancer Epidemiol. Biomarkers Prev..

[80] Li X., Xu H., Gao P. (2019). Fibrosis Index Based on 4 Factors (FIB-4) Predicts Liver Cirrhosis and Hepatocellular Carcinoma in Chronic Hepatitis C Virus (HCV) Patients. Med Sci. Monit..

[81] Bräu N., Fox R.K., Xiao P., Marks K., Naqvi Z., Taylor L.E., Trikha A., Sherman M., Sulkowski M.S., Dieterich D.T. (2007). Presentation and outcome of hepatocellular carcinoma in HIV-infected patients: A U.S.–Canadian multicenter study. J. Hepatol..

[82] Azam F., Latif M.F., Farooq A., Tirmazy S.H., Alshahrani S., Bashir S., Bukhari N. (2019). Performance Status Assessment by Using ECOG (Eastern Cooperative Oncology Group) Score for Cancer Patients by Oncology Healthcare Professionals. Case Rep. Oncol..

[83] Izzo F., Granata V., Grassi R., Fusco R., Palaia R., Delrio P., Carrafiello G., Azoulay D., Petrillo A., Curley S.A. (2019). Radiofrequency Ablation and Microwave Ablation in Liver Tumors: An Update. Oncology.

[84] Llovet J.M., Ricci S., Mazzaferro V., Hilgard P., Gane E., Blanc J.-F., De Oliveira A.C., Santoro A., Raoul J.-L., Forner A. (2008). Sorafenib in Advanced Hepatocellular Carcinoma. N. Engl. J. Med..

[85] Di Benedetto F., De Ruvo N., Berretta M., Masetti M., Montalti R., Di Sandro S., Quintini C., Codeluppi M., Tirelli U., Gerunda G.E. (2006). Don’t Deny Liver Transplantation to HIV Patients with Hepatocellular Carcinoma in the Highly Active Antiretroviral Therapy Era. J. Clin. Oncol..

[86] Chelis L., Ntinos N., Souftas V., Deftereos S., Xenidis N., Chamalidou E., Maltezos E., Kakolyris S. (2011). Complete response after sorafenib therapy for hepatocellular carcinoma in an HIV-HBV co infected patient: Possible synergy with HAART? A case report. Med Oncol..

[87] Perboni G., Costa P., Fibbia G.C., Morandini B., Scalzini A., Tagliani A., Cengarle R., Aitini E. (2010). Sorafenib Therapy for Hepatocellular Carcinoma in an HIV–HCV Coinfected Patient: A Case Report. Oncology.

[88] De Nardo P., Viscione M., Corpolongo A., Bellagamba R., Vennarecci G., Ettorre G.M., Gentilotti E., Tommasi C., Nicastri E. (2012). Treatment of Recurrent Hepatocellular Carcinoma with Sorafenib in a HIV/HCV Co-Infected patient in HAART: A Case Report. Infect. Agents Cancer.

[89] Ozenne V., Gervais A., Peytavin G., Castelnau C., Valla D.C., Degos F. (2011). Suspected interaction between sorafenib and HAART in an HIV-1 infected patient: A case report. Hepatogastroenterology.

[90] Berretta M., Di Benedetto F., Maso L.D., Cacopardo B., Nasti G., Facchini G., Bearz A., Spina M., Garlassi E., De Re V. (2013). Sorafenib for the treatment of unresectable hepatocellular carcinoma in HIV-positive patients. Anti-Cancer Drugs.

[91] Feng Y., Wang L., Lv H., Shi T., Xu C., Zheng H., Qi J., Zhao X., Li J., Gao Y. (2021). Microwave ablation versus radiofrequency ablation for perivascular hepatocellular carcinoma: A propensity score analysis. HPB.

[92] An C., Li W.-Z., Huang Z.-M., Yu X.-L., Han Y.-Z., Liu F.-Y., Wu S.-S., Yu J., Liang P., Huang J. (2021). Small single perivascular hepatocellular carcinoma: Comparisons of radiofrequency ablation and microwave ablation by using propensity score analysis. Eur. Radiol..

[93] Kim D.K., Han K., Won J.Y., Kim G.M., Kwon J.H., Kim M.D. (2020). Percutaneous cryoablation in early stage hepatocellular carcinoma: Analysis of local tumor progression factors. Diagn. Interv. Radiol..

[94] Cha S.Y., Kang T.W., Min J.H., Song K.D., Lee M.W., Rhim H., Lim H.K., Sinn D.H., Park M.S. (2019). RF Ablation Versus Cryoablation for Small Perivascular Hepatocellular Carcinoma: Propensity Score Analyses of Mid-Term Outcomes. Cardiovasc. Interv. Radiol..

[95] Wang F., Ma J., Wu L., Li N., Luo R., Wei G., Yang J., Yang J. (2020). Percutaneous cryoablation of subcapsular hepatocellular carcinoma: A retrospective study of 57 cases. Diagn. Interv. Radiol..

[96] Yang Y., Zhang Y., Wu Y., Chen J., Liang B., Chen Q., Wang Q., Lyu J., Li Y., Mu F. (2020). Efficacy and Safety of Percutaneous Argon-Helium Cryoablation for Hepatocellular Carcinoma Abutting the Diaphragm. J. Vasc. Interv. Radiol..

[97] Qi C., Gao H., Zhao Q., Zhang L. (2020). Computed Tomography-Guided Percutaneous Cryoablation for Subcardiac Hepatocellular Carcinoma: Safety, Efficacy, Therapeutic Results and Risk Factors for Survival Outcomes. Cancer Manag. Res..

[98] Mafeld S., Wong J.J., Kibriya N., Stenberg B., Manas D., Bassett P., Aslam T., Evans J., Littler P. (2018). Percutaneous Irreversible Electroporation (IRE) of Hepatic Malignancy: A Bi-institutional Analysis of Safety and Outcomes. Cardiovasc. Interv. Radiol..

[99] Stillström D., Beermann M., Engstrand J., Freedman J., Nilsson H. (2019). Initial experience with irreversible electroporation of liver tumours. Eur. J. Radiol. Open.

[100] Kalra N., Gupta P., Gorsi U., Bhujade H., Chaluvashetty S.B., Duseja A., Singh V., Dhiman R.K., Chawla Y.K., Khandelwal N. (2019). Irreversible Electroporation for Unresectable Hepatocellular Carcinoma: Initial Experience. Cardiovasc. Interv. Radiol..

[101] Colquhoun S.D. (2020). Hepatocellular carcinoma clinical update: Current standards and therapeutic strategies. Liver Res..

[102] Freeman E., Cheung W., Kavnoudias H., Majeed A., Kemp W., Roberts S.K. (2021). Irreversible Electroporation for Hepatocellular Carcinoma: Longer-Term Outcomes at A Single Centre. Cardiovasc. Interv. Radiol..

[103] Verloh N., Jensch I., Lürken L., Haimerl M., Dollinger M., Renner P., Wiggermann P., Werner J.M., Zeman F., Stroszczynski C. (2019). Similar complication rates for irreversible electroporation and thermal ablation in patients with hepatocellular tumors. Radiol. Oncol..

[104] Sugimoto K., Kakimi K., Takeuchi H., Fujieda N., Saito K., Sato E., Sakamaki K., Moriyasu F., Itoi T. (2019). Irreversible Electroporation versus Radiofrequency Ablation: Comparison of Systemic Immune Responses in Patients with Hepatocellular Carcinoma. J. Vasc. Interv. Radiol..

[105] Niessen C., Beyer L., Haimerl M., Schicho A., Stroszczynski C., Wiggermann P., Jung E. (2019). Percutaneous irreversible electroporation of hepatocellular carcinoma: Contrast-enhanced ultrasound-findings during 1-year follow-up. Clin. Hemorheol. Microcirc..

[106] Sundram F.X., Buscombe J.R. (2017). Selective internal radiation therapy for liver tumours. Clin. Med..

[107] Liu X., Wang Z., Chen Z., Liu L., Ma L., Dong L., Zhang Z., Zhang S., Yang L., Shi J. (2018). Efficacy and Safety of Transcatheter Arterial Chemoembolization and Transcatheter Arterial Chemotherapy Infusion in Hepatocellular Carcinoma: A Systematic Review and Meta-Analysis. Oncol. Res. Featur. Preclin. Clin. Cancer Ther..

[108] Kim J.H., Yoon H.-K., Kim S.Y., Kim K.M., Ko G.-Y., Gwon D.I., Sung K.-B. (2009). Transcatheter arterial chemoembolization vs. chemoinfusion for unresectable hepatocellular carcinoma in patients with major portal vein thrombosis. Aliment. Pharmacol. Ther..

[109] Nishikawa H., Osaki Y., Kita R., Kimura T., Ohara Y., Takeda H., Sakamoto A., Saito S., Nishijima N., Nasu A. (2013). Comparison of transcatheter arterial chemoembolization and transcatheter arterial chemotherapy infusion for patients with intermediate-stage hepatocellular carcinoma. Oncol. Rep..

[110] Soydal C., Arslan M.F., Kucuk O.N., Idilman R., Bilgic S. (2016). Comparison of survival, safety, and efficacy after transarterial chemoembolization and radioembolization of Barcelona Clinic Liver Cancer stage B-C hepatocellular cancer patients. Nucl. Med. Commun..

[111] Gordon A., Lewandowski R., Hickey R., Kallini J., Gabr A., Sato K., Desai K., Thornburg B., Gates V., Ganger D. (2016). Prospective randomized phase 2 study of chemoembolization versus radioembolization in hepatocellular carcinoma: Results from the PREMIERE trial. J. Vasc. Interv. Radiol..

[112] Kolligs F.T., Bilbao J.I., Jakobs T., Iñarrairaegui M., Nagel J.M., Rodriguez M., Haug A., D’Avola D., Winkel M.O.D., Martinez-Cuesta A. (2015). Pilot randomized trial of selective internal radiation therapy vs. chemoembolization in unresectable hepatocellular carcinoma. Liver Int..

[113] Reid E., Suneja G., Ambinder R.F., Ard K., Baiocchi R., Barta S.K., Carchman E., Cohen A., Gupta N., Johung K.L. (2018). Cancer in People Living With HIV, Version 1.2018, NCCN Clinical Practice Guidelines in Oncology. J. Natl. Compr. Cancer Netw..

[114] Di Benedetto F., De Ruvo N., Berretta M., Masetti M., Montalti R., Di Sandro S., Ballarin R., Codeluppi M., Guaraldi G., Gerunda G. (2008). Hepatocellular carcinoma in HIV patients treated by liver transplantation. Eur. J. Surg. Oncol..

[115] Di Benedetto F., Di Sandro S., De Ruvo N., Berretta M., Montalti R., Guerrini G.P., Ballarina R., De Blasiis M.G., Spaggiari M., Smerieri N. (2008). Human Immunodeficiency Virus and Liver Transplantation: Our Point of View. Transplant. Proc..

[116] Ettorre G.M., Vennarecci G., Boschetto A., Giovannelli L., Antonini M., Carboni F., Santoro R., Lepiane P., Cosimelli M., Lonardo M.T. (2003). Resection and transplantation: Evaluation of surgical perspectives in HIV positive patients affected by end-stage liver disease. J. Exp. Clin. Cancer Res..

[117] Xia Y.X., Zhang F., Li X.C., Kong L.B., Zhang H., Li D.H., Cheng F., Pu L.Y., Zhang C.Y., Qian X.F. (2021). Surgical treatment of primary liver cancer:a report of 10 966 cases. Zhonghua Wai Ke Za Zhi.

[118] Kong L., Wei G., Lv T., Jiang L., Yang J., Zhao Y. (2021). Outcome of TACE treatment in HIV infected patients with hepatocellular carcinoma. Sci. Rep..

[119] Lim C., Goutte N., Gervais A., Vullierme M.-P., Valla D., Degos F., Farges O. (2012). Standardized Care Management Ensures Similar Survival Rates in HIV-Positive and HIV-Negative Patients with Hepatocellular Carcinoma. JAIDS J. Acquir. Immune Defic. Syndr..

[120] Berretta M., Garlassi E., Cacopardo B., Cappellani A., Guaraldi G., Cocchi S., De Paoli P., Lleshi A., Izzi I., Torresin A. (2011). Hepatocellular Carcinoma in HIV-Infected Patients: Check Early, Treat Hard. Oncology.

